# Turning Down the Volume: Why Some Genes Tolerate Less Noise

**DOI:** 10.1371/journal.pbio.0020191

**Published:** 2004-04-27

All organisms have evolved complex mechanisms designed to exquisitely regulate the expression of appropriate genes at their correct levels. Natural random variation in the processes of regulation and expression, however, limits the precision with which protein production can be controlled. This subtle variation, or “noise,” in the expression of genes has been studied with increasing interest. Though much progress has been made in understanding the amount of noise that exists and the cellular processes that underlie it, the physiological impact of noise, and whether it is biologically relevant or can just be ignored, has been less clear.[Fig pbio-0020191-g001]

**Figure pbio-0020191-g001:**
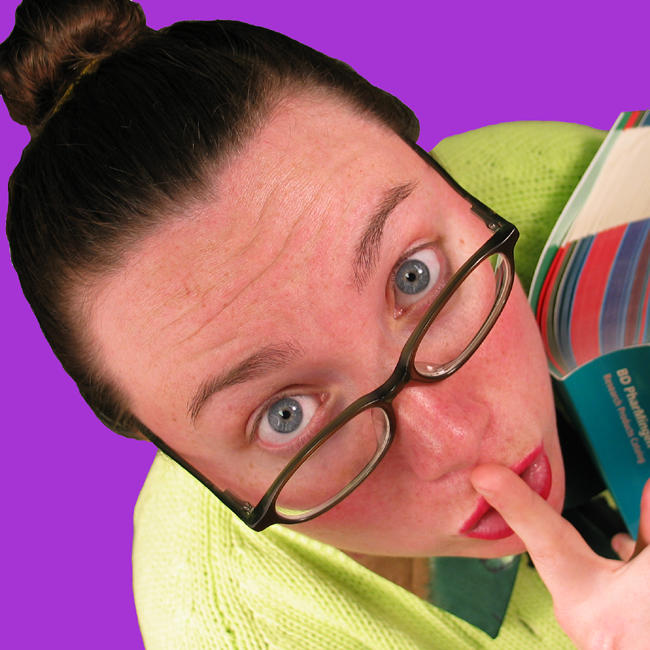
“Shhhhhhhhhhhhhh!” (Photo by Bryan Zeitler and Jennifer Zeitler)

To answer this question, Hunter Fraser et al. asked whether noise in gene expression exerts an equal effect on all genes in the genome. Is noise in gene expression irrelevant to the fitness and well-being of cells, or do cells need to minimize noise in the expression of some or all genes? If noise in gene expression has a negative impact on cells, they reasoned, that impact should vary from gene to gene depending on the gene's function. There should be selection to minimize noise for those genes most crucial to cell survival and function. Thus, a genome-wide analysis of noise in gene expression, they predicted, would show that genes for which “noisy” expression would be most harmful would display less of it.

The researchers examined this question in the budding yeast Saccharomyces cerevisiae because of the vast quantity and variety of genomic data available for this organism. Previous research has shown that the noise that exists in the expression of a gene is directly related to the rates of transcription and translation. Using data available from previous genome-wide studies, the authors were able to estimate these rates, and therefore the noise, for nearly every gene in the yeast genome.

After estimating the amount of noise in the expression level for nearly every gene, the authors examined two subsets of genes that they hypothesized would be particularly affected by noise. First they looked at “essential” genes, reasoning that since total lack of expression of these genes results in death, even small variations in expression resulting from noise would often exert a negative impact. Previous research had identified all the essential genes in yeast by deleting each gene individually and assessing the fitness of the resulting mutant. Here, the authors compared the levels of noise in this pool of essential genes to that of nonessential genes. They found that essential genes usually display less noise than nonessential genes, lending support to their hypothesis.

They similarly examined genes encoding proteins involved in forming multiprotein complexes. Because these complexes are built of proteins in specific ratios, over- or under expression of one component will hinder the accurate assembly of productive complexes. So a high degree of noise could interfere with the coordinated expression necessary for proteins involved in these complexes. The authors again used data from previous research to choose members of this group: they relied on two studies which had identified a large number of multiprotein complexes in yeast. Using this group of genes for comparison, the authors found that, like essential genes, genes encoding proteins involved in multiprotein complexes generally display less noise than other genes.

This study draws a simple but fundamental conclusion about noise in eukaryotic gene expression—noise has physiological consequences. Importantly, the fact that noise is minimized in those gene groups for which noisy expression would be most harmful suggests that factors contributing to noise are subject to natural selection. This study also demonstrates the power of using the growing number of genomescale datasets in this type of analysis. Researchers will undoubtedly continue to mine the available data to draw biological conclusions not anticipated by the original authors.

